# *In-vitro* Activity of *Trigonella foenum graecum *Seeds Against a Clinical Strain of *Acanthamoeba* Genotype T4

**Published:** 2018

**Authors:** Samira Dodangeh, Maryam Niyyati, Mohammad Kamalinejad, Jacob Lorenzo-Morales, Abdolali Moshfe, Ali Haghighi, Eznolah Azargashb

**Affiliations:** a *Cellular and Molecular Biology Research Center, Shahid Beheshti University of Medical Sciences, Tehran, Iran. *; b *Traditional Medicine and materia Medica Research Center, Shahid Beheshti University of Medical Sciences, Tehran, Iran. *; c *Department of Medical Parasitology and Mycology, School of Medicine, Shahid Beheshti University of Medical Sciences, Tehran, Iran. *; d *School of Pharmacology, Shahid Beheshti University of Medical Sciences, Tehran, Iran. *; e *University Institute of Tropical Diseases and Public Health of the Canary Islands, University of La Laguna, Tenerife, Canary Islands, Spain. *; f *Cellular and Molecular Research Center, Yasuj University of Medical Sciences, Yasuj, Iran. *; g *Department of Family Medicine, School of Medicine, Shahid Beheshti University of Medical Sciences, Tehran, Iran.*

**Keywords:** Acanthamoeba, Trigonella foenum graecum, seeds, aqueous extract, in-vitro

## Abstract

*Acanthamoeba *keratitis (AK) is a sight-threatening infection of the cornea disease that often presents with a lengthy and not fully effective treatment. Current therapeutic options against *Acanthamoeba *are not very effective against the cyst. Calibrated trophozoite/cyst suspension was incubated with the same volume of serial dilutions of the *Trigonella foenum graecum* aqueous extract (200, 250, 350, 450, 600, and 750 mg/mL) in microcentrifuge tubes and mixed by pipetting up and down. After that, the tubes were incubated at 26 ºC for 24, 48, and 72 h. The obtained result revealed that incubation of the extract (at concentrations ranging from 200 to 750 mg/mL) with *Acanthamoeba* was able to decrease the number of viable trophozoites and cysts. In the presence of up to 450 mg/mL non-viable trophozoites were observed whereas cysts were only eliminated when incubated with 750 mg/mL of the extract after 24 h. Furthermore, no cytotoxicity of the extract even at the highest concentration tested in the study showed to be toxic for corneal cells. Further studies should be carried out in order to elucidate the active compounds involved in the observed anti-*Acanthamoeba* activities which could be used for the development of novel therapeutic approaches against *Acanthamoeba* infections.

## Introduction

Free-living amoebae of *Acanthamoeba* genus are ubiquitous protozoa widely distributed in nature and often isolated from soil and water sources. Pathogenic strains of *Acanthamoeba* are causative agents of lethal encephalitis and a sight-threating infection of the cornea known as *Acanthamoeba* keratitis (AK). Contact lens wearers are at increased risk of contracting AK with studies estimating that around 90% of AK patients are users of contact lenses (1, 2). Moreover, inadequate contact lens hygiene practices and the use of contaminated tap water and home-made saline solutions for cleaning the lenses and storage cases are the main risk factors for contacting AK (2-4). Clinical symptoms of AK include photophobia, severe pain, epithelial cell loss, a characteristic stromal ring-like infiltrate and blurred vision (2, 5). Regarding AK, it is important to mention that the lack of standardized diagnostic tests and awareness among clinicians results in a late diagnosis. Moreover, effective treatment with a combination of drugs has been reported mostly when initiated in early stages of the disease. However, when diagnosis is late, the majority of current therapeutic agents have been reported to be ineffective since amoebic cysts resist the action of these agents within the corneal stroma (4). 

Currently, the recommended treatment regimen for AK includes a biguanide (0.02% polyhexamethylene biguanide, PHMB, or 0.02% chlorhexidine digluconate) together with a diamidine (0.1% propamidine isethionate, also known as Brolene, or 0.1% hexamidine, also known as Desomedine) (6). Chlorhexidine and PHMB as monotherapy agents have been proven not to be sufficient against clinical or environmental strains of *Acanthamoeba* and have highlighted the importance of multiple-strain testing of drugs against *Acanthamoeba* as their effectiveness might depend on the *Acanthamoeba* isolate (6-8). 

Higher plants are currently used as natural sources for the discovery of novel active compounds (9). Many other natural products of diverse molecular structure have demonstrated anti-parasitic activity in the laboratory and represent interesting lead structures for the development of new and urgently needed anti-parasitic drugs (10). The herb *Trigonella foenum graecum *(commonly known as Fenugreek) belongs to the *Fabaceae *(Papilionaceae) family and is grown in Iran, India, Northern Africa, and the United States (11). Interestingly, it is used in folk medicine in Iran (“Shanbalileh” in Iranian traditional medicine) due to its hypoglycaemic and anti-rheumatic properties (12, 13). 

Previous studies on this plant species have demonstrated its activity against *Plasmodium falciparum *(14). Moreover, phytochemical screening of *Trigonella foenum graecum* has shown the presence of flavonoids, saponins, and alkaloids among other bioactive molecules (5, 15, 16). 

In the present study, the *in-vitro* activity of the aqueous extract of *Trigonella foenum graecum *seeds was evaluated against trophozoites and cysts of a clinical strain of *Acanthamoeba*. Additionally, an evaluation of its cytotoxic potential on corneal cells was also performed. 

## Experimental

*Preparation of the aqueous extract of Trigonella foenum graecum seeds*

Specimens of this plant species were collected from the Ray area of Iran and identified and approved by a local herbalist (Mohammad Kamalinejad) from the School of Pharmacology of Shahid Beheshti University of Medical Sciences, Tehran, Iran). The aqueous extract of the seeds was prepared, mixing 2 L of boiling distilled water per 100g of dried seeds of *Trigonella foenum graecum*. After that, the pan was quickly removed from the heat, covered with foil and after 4 h the contents of the container were squeezed and filtered using a 0.22 µm filter (Whatman paper). The obtained extract was dried for 24 h in a water bath in order to fully evaporate the water (17). 

*Acanthamoeba* *strain *

The *Acanthamoeba* strain tested in this study was previously isolated from a corneal scrape which was collected from a soft contact lens wearer affected with AK. Briefly, the specimen was inoculated onto the surface of 1.5% non-nutrient agar (NNA) plates coated with heat killed *Escherichia coli *and incubated at 26 °C for up to 72 h. Following DNA extraction, PCR was used to confirm the microscopic identification. PCR analysis, DNA sequencing, and BLAST analysis allowed the classification of this strain as a member of the T4 genotype. 

*Trophozoites preparation*

*Acanthamoeba *was grown at 26 °C in PYG axenic medium (0.75% [wt/vol] proteose peptone, 0.75% [wt/vol] yeast extract, and 1.5% [wt/vol] glucose) according to a previous study (8, 18). One mL of trophozoites in the stage of exponential growth (72–96 h) was collected and centrifuged for 5 min at 2000 g, the supernatant discarded, and the sediment was washed twice with phosphate-buffered saline buffer (PBS). The viability of trophozoites was determined using 1% eosin and cell counts were done using a cell-counter chamber. Final concentration was adjusted to 25 × 10^4 ^trophozoites per mL for the activity assays (19, 20). 

*Cysts preparation*

One hundred microliter of the axenic medium was inoculated directly onto the surface of 1.5% non-nutrient agar (NNA) plates and incubated in a humidified chamber at 26 °C. The agar surface was covered with 5 mL of PBS and cysts were removed from the base of NNA culture plates using a sterile cell scraper. Cysts were harvested from the suspension by centrifugation at 1500 g for 5 min, the supernatant was discharged, and the pellet was washed twice with PBS. Cysts in the obtained suspension were counted with a cell-counter chamber, and the suspension was adjusted to 25×10^4 ^cysts/mL for the cysticidal activity assays (20, 21). 

*Evaluation of the activity*

150 µL of the calibrated trophozoite/cyst suspension was incubated with the same volume of serial dilutions of the *Trigonella foenum graecum* aqueous extract (200, 250, 350, 450, 600, and 750 mg/mL) in microcentrifuge tubes and mixed by pipetting up and down. After that, the tubes were incubated at 26 °C for 24, 48, and 72 h. In addition, control tubes were prepared: a negative control with trophozoites/cysts and PBS and a positive control with trophozoite/cysts and 0.02% chlorhexidine digluconate (prepared from a solution 20% in water CHX, C-9394; Sigma).

Three tubes were prepared for the evaluation of each concentration and measurements were repeated 5 times (22). 

*Effects of the extract against trophozoites and cysts of Acanthamoeba*

After the incubation periods at 26 °C, 25 µL of the amoebic suspensions were mixed with the same volume of 1% eosin in a counting chamber. Unstained (viable) and stained (non-viable) cells were then counted. Approximately 100 *Acanthamoeba* trophozoites were examined in each time and all the tests were repeated three times. Additionally, cultures containing non-viable cysts were transferred onto a NNA agar plates seeded with *E. coli* and incubated at 26 °C during three days to confirm the observed results (22).

**Table 1:** Effect of *Trigonella foenum graecum *aqueous extract on the survival and growth of *Acanthamoeba *trophozoites and cysts

72 h	**48** h	24 h	Experimental periods	Amoebae form	Dose (mg/mL)
**0** ** ± 0**	0 ± 0	0 ± 0^*^		Trophozoites	750
**0** ** ± 0**	0 ± 0	0 ± 0		Cysts	
**0** ** ± 0**	0 ± 0	0 ± 0		Trophozoites	600
**0** ** ± 0**	0 ± 0	0.4 ± 0.63		Cysts	
**0** ** ± 0**	0 ± 0	0 ± 0		Trophozoites	450
**0** ** ± 0**	3.07 ±1.22	4.0 ± 1.13		Cysts	
**0.87 ± 0.51**	1.0 ± 0.53	3.07 ±1.22		Trophozoites	350
**1.0 ± 0.53**	6.47 ± 1.24	7.7 ±1.38		Cysts	
**4.1 ± 1.12**	4.0± 1.13	4.8 ± 1.14		Trophozoites	250
**4.0 ± 1.13**	9.07 ± 2.54	10.07 ± 2.08		Cysts	
**5.0 ±.1.41**	5.0 ± 1.41	8.07± 1.27		Trophozoites	200
**5.0±1.41**	13.0 ± 1.64	15.0 ± 2.1		Cysts	
**22.53±1.69**	23.7± 1.62	24.67± 2.05		Trophozoites	Control
**23.5±1.75**	23.07±1.62	24.71± 2.05		Cysts	

* Data were expressed as mean ± SD.

**Table 2:** Cytotoxic effect of *Trigonella foenum graecum* on corneal cells using MTT.

***Trigonella foenum graecum*** ** (mg/mL)**	**% Cell viability**
200	10
250	100
350	100
450	100
600	100
750	99
850	95
1000	94
1200	93
1500	93
2000	90
Control	100

**Figure 1:**
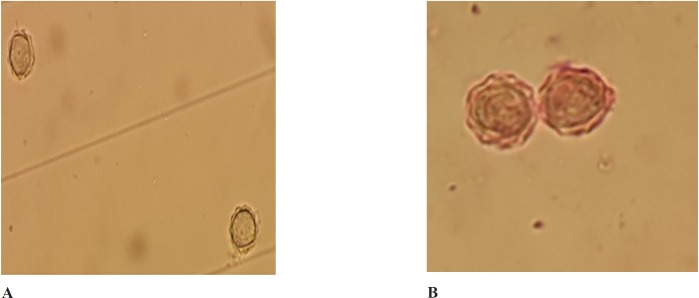
A. Effect of aqueous extract of *Trigonella foenum graecum* on the proliferation of pathogenic *Acanthamoeba *cysts before treatment.B: Effect of aqueous extract of *Trigonella foenum graecum* on the proliferation of pathogenic *Acanthamoeba* cysts after treatment (at 750 mg/mL concentration (72 h

*Cell line and culture conditions*

Human corneal epithelial cells (HCEC) (Invitrogen) were grown in Defined Keratinocyte SFM medium (DKSFM) according to manufacturer’s instructions. Cells were prepared at 90% confluence for the cytotoxicity assays.

*Evaluation of the toxicity of the aqueous extract*

The corneal cells (4 ×10^4^ cell per well) were incubated in 96 well plates for 24 h in the presence and absence of the tested concentrations of the plant extract. After that, twenty microliters of a MTT solution (MTT assay (4,5-dimethylthiazol-2-yl)-2, 5-diphenyl tetrazolium bromide) were added to each well. The plates were then incubated for 4 h at 37 °C in a CO_2_ incubator. In the next step 180 µL of medium was discarded and 180 μL of methanol/DMSO solution (50:50) were added to each well. Plates were then further incubated on a plate shaker after adding formazan crystals to allow thoroughly mixture of the added reagents. The cells viability was checked using colorimetric assay with micro plate reader (Biotek, Germany) (23).

The statistical analysis of data was performed using the SPSS software version 15.0. *P* values < 0.001 were considered as statistically highly significant.

## Results

The present study was investigated the amoebicidal activity of the aqueous extract of the seeds of *Trigonella foenum graecum*. The obtained results showed that the tested extract exhibited activity against the trophozoite stage of *Acanthamoeba* at concentrations ranging from 200 to 750 mg/mL. The lowest concentration of the extract that was tested (200 mg/mL) failed to eliminate the whole population of trophozoites. Furthermore, cysts were only eliminated when incubated (for 24 h) with concentrations of 750 mg/mL of the aqueous extract (Table 1). In the case of 600 mg/mL, only 1.6% of cysts wasn’t affected after 24 h, however non-viable cysts were observed after 48 h. At the concentration of 750 mg/mL, all cysts were eliminated during the duration of the experiment (Table 1). Light microscopy revealed that the untreated *Acanthamoeba* cyst contained double walls: the ectocyst and endocyst walls. The endocyst appeared smooth and a clear space separated it from wrinkled ectocyst and non-treated trophozoite showed acanthopodia. Cysts treated with the extract showed a cytoplasmic clump and rounded walls and even some cysts presented empty walls (Figure 1A and 1B).

Nevertheless, it is important to mention that a time and dose dependent amoebicidal action of the extract was shown on both stages of the amoebae. In conclusion, in the presence of up to 450 mg/mL of the aqueous extract, non-viable trophozoites were observed whereas cysts were only eliminated when incubated with 750 mg/mL of the extract after 24 h. Furthermore, no cytotoxicity of the extract even at the highest concentration tested in the study showed to be toxic for corneal cells (Table 2). 

## Discussion

*Acanthamoeb*a keratitis has generally been a medical challenge to most ophthalmologists. This devasting corneal disease is often treated with a combination of drugs such as polyhexamethylene biguanide (PHMB) or chlorhexidine and propamidine isethionate. However, only half of the patients have been reported to improve after treatment with the mentioned drugs when the disease is not early diagnosed (24). Moreover, biguanides present toxic effects on human keratocytes even at the lowest cysticidal concentrations (25). Treatment course for the mentioned drugs is also very lengthy and it may even last up to six months (26). The main drawback to the mentioned drugs is their toxicity and poor cysticidal effect (27, 28). 

Thus, cysts resistance to current available drugs has prompted the researchers to develop novel anti-amoebic compounds. Recently, there is a raising trend to shift resources from currently chemical drugs to medicinal plants (29). Indeed, finding a natural compound with amoebicidal and cysticidal effect and non-toxic to human cells is of utmost importance to manage AK cases. 

In our study, the activity of the aqueous extract of *Trigonella foenum graecum* seed was evaluated against a clinical strain of *Acanthamoeba *since a previous study reported that this medicinal plant presented anti-parasitic properties when assayed in *Leishmania donovani *and *Plasmodium falciparum *(16, 30). The present study showed that the tested aqueous extracts were able to eliminate trophozoites and cysts of *Acanthamoeba *at the tested concentrations. It should be mentioned that in comparison with other researches regarding other medicinal plants, we tested a higher concentrations (up to 750 mg/mL) and accordingly cytotoxic evaluation on corneal cells was carried out. The results confirmed that *Trigonella foenum graecum* extracts has no cytotoxic effect on the culture of corneal cells with the dose of 750 mg/mL.

In addition, *T. foenum graecum *is a widely used medicinal plant presenting antidiabetic, anti-inflammatory, antipyretic (31)_, _anthelmintic, and antibacterial properties (32). On the other hand, previous studies revealed that oral consumption of *Trigonella foenum graecum *lead to significant protection against eye cataract *in-vivo *(33). In an animal study, *T. foenum graecum* seeds powder showed the acute oral LD50 (Lethal Dosage) of > 5 g/kg in rats (34). Further studies should be carried out in order to identify the active compounds in *T. foenum graecum* seeds.

Overall, the obtained experimental results of the present research demonstrated that *T. foenum graecum* could be used in the near future in order to obtain amoebicidal and cysticidal compounds for the treatment of *Acanthamoeba* infections. Toxicity tests reflected that the mentioned plant have none side-effects in corneal cells. Further *in-vitro *and *in-vivo* studies are needed in order to elucidate the active compounds involved in the observed anti-*Acanthamoeba* activities which could be used for the development of novel therapeutic approaches against *Acanthamoeba *infections.
